# Micellar Enhanced Spectrofluorimetric Method for the Determination of Ponatinib in Human Plasma and Urine via Cremophor RH 40 as Sensing Agent

**DOI:** 10.1155/2015/210503

**Published:** 2015-12-31

**Authors:** Hany W. Darwish, Ahmed H. Bakheit, Ali Saber Abdelhameed, Amer S. AlKhairallah

**Affiliations:** ^1^Department of Pharmaceutical Chemistry, College of Pharmacy, King Saud University, P.O. Box 2457, Riyadh 11451, Saudi Arabia; ^2^Analytical Chemistry Department, Faculty of Pharmacy, Cairo University, Kasr El-Aini Street, Cairo 11562, Egypt; ^3^Quality Management Department, National Guard Health Affairs, King Abdulaziz Medical City, P.O. Box 22490, Riyadh 11426, Saudi Arabia

## Abstract

An impressively simple and precise spectrofluorimetric procedure was established and validated for ponatinib (PTB) quantitation in biological fluids such as human plasma and human urine. This method depends on examining the fluorescence characteristics of PTB in a micellar system of Cremophor RH 40 (Cr RH 40). Cr RH 40 enhanced the intrinsic fluorescence of PTB distinctly in aqueous water. The fluorescence spectra of PTB was recorded at 457 nm following its excitation at 305 nm. Maximum fluorescence intensity was attained by addition of 0.7 mL of Cr RH 40 and one mL of phosphate buffer to PTB aliquots and then dilution with distilled water. There is a linear relationship between the fluorescence intensity of PTB and its concentration over the range 5–120 ngmL^−1^, with limit of detection and limit of quantification equal to 0.905 ngmL^−1^ and 2.742 ngmL^−1^, respectively. The accuracy and the precisions of the proposed method were checked and gave adequate results. The adopted method was applied with a great success for PTB quantitation in different biological matrices (spiked human plasma and urine) giving high recovery values.

## 1. Introduction

**Figure 1:**
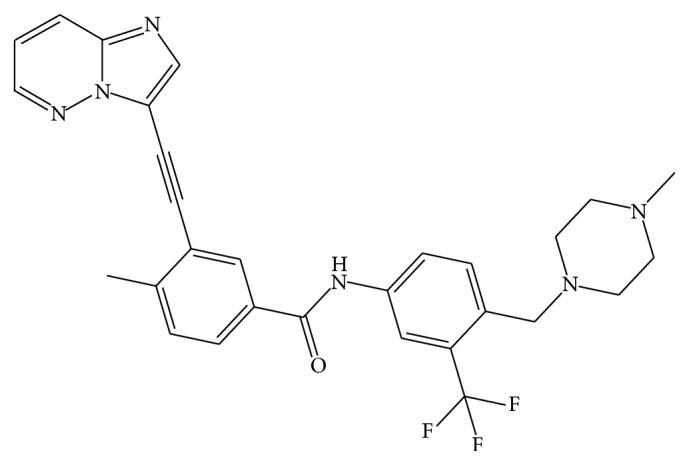
Chemical structure of ponatinib (PTB).

Ponatinib (PTB; Figure 1) is an orally bioavailable drug established for management of chronic Philadelphia chromosome-positive (Ph+) acute lymphoblastic leukemia (ALL) and myeloid leukemia (CML) [1].

The BCR-ABL oncogene, which is the result of Philadelphia chromosome (Ph) 22q, encodes a chimeric BCR-ABL protein which is responsible for the activity of ABL tyrosine kinase. This in turn is the basic reason of chronic myeloid leukaemia (CML) [2]. ABL is “Abelson” gene on chromosome 9, while BCR is “breakpoint cluster region” gene on chromosome 22.  For the past few years, different trials were made to develop a treatment for cases with CML who have become unresponsive to the different TKIs available drugs, such as imatinib and dasatinib. Ponatinib is particularly more effective than the other TKIs because it can treat patients who exhibit a T315I mutation [3].

United States Food and Drug Administration (US FDA), on December 2012, approved PTB tablets (Iclusig tablets, manufactured by ARIAD Pharmaceuticals, Inc.) for the management of adult patients who suffered from accelerated, chronic, or blast-phases chronic myeloid leukemia (CML) or Philadelphia chromosome-positive acute lymphoblastic leukemia (Ph+ ALL) which is unaffected by the previous tyrosine kinase inhibitors [4, 5]. On October 2013, US FDA and ARIAD Pharmaceuticals, Inc., decided to hang up marketing of PTB. This was based on the remarkable increment in the cases of severe arterial thrombosis occasions recognized by continuous monitoring of the drug by the USFDA [5, 6]. In January 2014, the manufacturer recommenced marketing PTB with a warning clearly written on the box to spread caution from vascular occlusion and hepatotoxicity occurrence in patients receiving PTB [5, 7].

Usually a thorough understanding of relationships of medication levels with medication activity is crucial for the routine use of this medication. Therapeutic drug monitoring is based on accurate and precise measurements of the drug in biological samples, for example, blood and urine, at several times through the treatment.

Regarding patients, clinical assessments and additional treatment alternatives should be taken in light of precise validated analytical methods. In like manner, when determining the toxic level, efficacy, or pharmacokinetics of novel therapeutic agents and/or new drug combinations, efficient and reliable analytical techniques are needed. Consequently, comprehensive specific, accurate, and precise method to determine PTB in biological fluids is essential. On the other hand, fluorescence spectroscopy has been known to afford sufficiently efficient and reliable approach to determine numerous medications either in bulk form or in different matrices [8, 9]. The application of micelle-enhanced spectrofluorimetry has also been demonstrated to increase the sensitivity for the quantitation of several small molecules [10–12]. This is completely based on the capability to form micelle which in turn improves intensity of the compounds of weak fluorescence. Furthermore, micelle-enhanced spectrofluorimetric methods presented efficient and green chemistry approach, due to the absence of any organic solvent. These methods have been depended on surface active agents such as tween [13, 14], sodium dodecyl sulphate (SDS) [15, 16], and cyclodextrin [17, 18]. Nevertheless, our group recently was the first to report the use of the nonionic surface active agent “Cremophor RH 40; Cr RH 40” in enhanced micellar spectrofluorimetry [19, 20]. The manufacture of Cr RH 40 comprises reaction of forty moles of ethylene oxide and hydrogenated castor oil. Cremophor RH 40 is composed primarily of triricinoleate ester of ethoxylated glycerol and minor quantities of polyethylene glycol ricinoleate and the equivalent free glycols [21]. Upon literature review, there were no published analytical methods to quantify PTB in biological samples.

Thus, the current study is designed to establish sensitive and validated spectrofluorimetric method for the quantitation of PTB in spiked human plasma and urine. The proposed procedure was characterized by simplicity, sensitivity, and reproducibility.

## 2. Experimental

### 2.1. Instrument

Jasco FP-8200 Fluorescence Spectrometer (Jasco Corporation, Japan) equipped with a 150 W xenon lamp and 1 cm quartz cells was utilized for all fluorescence measurements. The slit widths for excitation and emission monochromators were adjusted at 5.0 nm. Calibration and linearity of the apparatus were regularly evaluated with standard solution of 0.01 *µ*gmL^−1^ quinine sulphate. Calibration of wavelength calibration was achieved by measuring *λ*_ex_ at 275 nm and *λ*_em_ at 430 nm; no wavelength variation was detected. SpectraManager software was used for changing the format of the recorded spectra to ASCII. For pH adjustments, Hanna pH-Meter (Romania) was utilized.

### 2.2. Reagents and Materials

The utilized solvents were of HPLC grade and all chemicals were of Analytical Reagents grade. Ponatinib reference powder with claimed purity of 99.6% was procured from LC Labs (Woburn, MA, USA). Cr RH40 and Cr EL procured from BASF (Ludwigshafen, Germany) and utilized as 1% v/v solution Cremophor RH40 and 1% v/v in water for Cremophor EL. SDS (Sodium dodecyl sulphate; 95%) was obtained from Winlab (Pontefract, London, UK) and prepared as 1% w/v in water. Β-CD (*β*-cyclodextrin) and CMC (carboxymethylcellulose) were both purchased from Merck (Darmstadt, Germany) and dissolved in water as 1% w/v. Tween 20, 80, and 85 were procured from Techno Pharmchem Haryana Company (New Delhi, India) and utilized as 1% v/v in water. Ethanol and methanol were from VWR Prolabo (Fontenay Sous Bois, France) and ACN (acetonitrile) was from Sigma-Aldrich Chemie GmbH (Schnelldorf, Germany). 0.1 M Phosphate buffer and 0.1 M borate buffer, covering the pH ranges 2–7 and 8–10, respectively, were freshly prepared, all reagents, namely, sodium hydroxide, boric acid, potassium chloride, phosphoric acid, disodium hydrogen phosphate, and potassium dihydrogen phosphate, were of spectroscopic grade. Ultrapure water was acquired through a Millipore Milli-Q UF Plus water purifier (MA, USA). Plasma samples were obtained from King Khaled University Hospital (KSU, Riyadh, Saudi Arabia). All patients provided written informed consent, then fasting blood specimens were collected followed by plasma separation and storage at −70°C.

### 2.3. Standard Solutions

A stock solution of PTB (1 mgmL^−1^) was prepared by dissolving 25 mg of PTB reference standard powder into 25 mL acetonitrile in a 25 mL measuring flask and diluting to the mark appropriately. The stock solution was then diluted two times with methanol to prepare a working standard solution of 1 *μ*gmL^−1^. The stability of these standard solutions were checked for at least 14 days when held in the refrigerator.

### 2.4. Calibration Graph Construction

Different samples were prepared by transferring portions of standard solutions of PTB to a set of five mL measuring flasks followed by 0.7 mL of Cr RH 40 and one mL of phosphate buffer. The volume was then completed using distilled water to obtain final concentrations of 5–120 ngmL^−1^. The flasks' contents were mixed well and the fluorescence intensity was recorded at 457 nm (after excitation at 305 nm). For construction of calibration graph, FI of the prepared samples was plotted againstcorresponding PTB concentration in ngmL^−1^. Ultimately, linear regression equation of the calibration curve was calculated.

### 2.5. Analysis of Human Plasma Samples

Twenty microliters of PTB standard solutions (representing variable PTB concentrations) was spiked each into a 1 mL aliquot of human plasma and mixed for 60 seconds to attain final concentrations of PTB of 50 ngmL^−1^, 60 ngmL^−1^, 70 ngmL^−1^, and 75 ngmL^−1^. Subsequently, a one milliliter volume of NaOH 100 mM/glycine buffer pH~11 was added and the tube was vortexed for 10 s. Liquid-liquid extraction was achieved using definite volume of diethyl ether (5 mL) and the solution was vortexed for 30 seconds followed by centrifugation for 15 minutes at 10,000 rpm to ensure complete phases separation. Four milliliters of the organic phase was pooled into glass vials and allowed to dry* via *a gentle stream of nitrogen. Finally, dry residue was reconstituted in ACN, followed by implementation of the general steps mentioned in Calibration Graph Construction. Treatment of a blank samples took place under similar conditions. FI was recorded at 457 nm (after excitation at 305 nm) and linear regression equation was applied for calculation of PTB concentration.

### 2.6. Analysis of Human Urine Samples

Spiking of one mL of human drug free urine took place with 20 *µ*L of different PTB standard solutions and mixed for 1 min. A one milliliter volume of NaOH 100 mM/glycine buffer pH~11 was added followed by mixing for 10 s. Liquid-liquid extraction was achieved using definite volume of diethyl ether (5 mL) and the solution was vortexed for 30 seconds followed by centrifugation for 15 minutes at 10,000 rpm to ensure complete phases separation. At that point four milliliters of the organic phase was pooled into glass vials and allowed to dry* via *a gentle stream of nitrogen. Lastly, the residue was reconstituted in ACN and appropriate dilutions were performed to yield final PTB concentrations of 10 ngmL^−1^, 20 ngmL^−1^, 60 ngmL^−1^, and 120 ngmL^−1^. Finally, the procedures termed under Calibration Graph Construction were accomplished. Treatment of a blank urine sample took place in a similar way. FI was recorded at 457 nm (after excitation at 305 nm) and linear regression equation was applied for calculation of PTB concentration.

## 3. Results and Discussion

It is essential to develop sensitive and reliable method for PTB quantitation in biological samples, for example, human plasma and human urine. Spectrofluorimetry is characterized by high sensitivity, enhanced selectivity, wide availability in quality control laboratories, and simplicity. These advantages were the motives for adopting this technique in our work. Experimental parameters that may influence the fluorescence intensity of PTB were tested and adjusted precisely, altering one parameter at a time while other parameters are held constant.

### 3.1. Fluorescence Spectra and Characteristics of PTB

**Figure 2:**
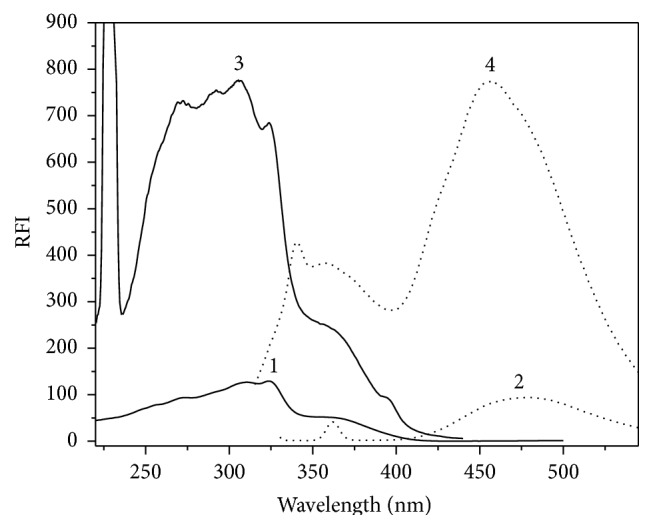
Fluorescence spectra representing (1) excitation and (2) emission of PTB in water (40 ngmL^−1^); (3) excitation and (4) emission (40 ngmL^−1^) of PTB in Cr RH 40 (1%, w/v).

Generation of fluorescence spectrum is based on absorption of electromagnetic radiation. PTB displays an excitation wavelength of 305 nm. PTB's, being a fluorescent compound, emission spectrum was recorded (utilizing a 1 *µ*g/mL) setting the excitation monochromator at 305 nm and scanning the emission monochromator in the range of 320–580 nm. An intensified emission peak with maximum at 457 nm occurred in the spectrum, demonstrating PTB fluorescence behaviour. The fluorescence spectra of PTB in aqueous medium and Cr RH 40 were tried (Figure 2).

Existence of Cr RH 40 gave rise to enhancement of PTB fluorescence intensity by nearly eightfold when compared with its intrinsic one in water. This improvement may be attributed to the difference in the microenvironment around PTB in micellar medium when compared to water, which may be due to limitations upheld on the unrestricted rotations that are in rivalry with emission [12].

### 3.2. Experimental Conditions' Optimization

#### 3.2.1. Influence of Organized Media

**Figure 3:**
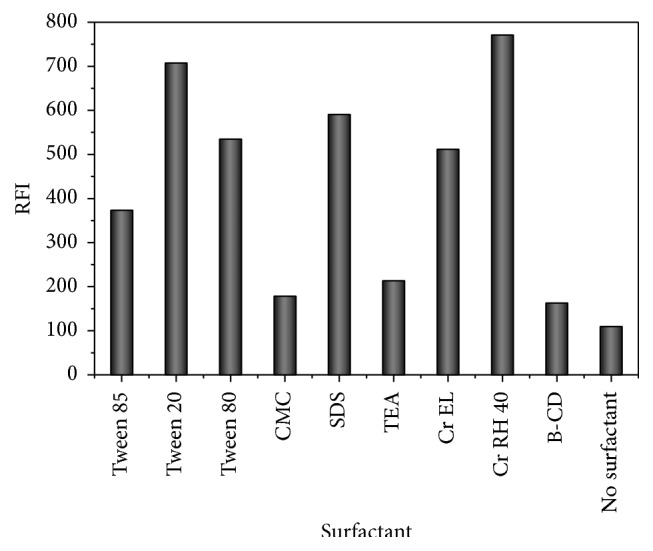
Effect of the type of organized media (0.5 mL, 1% w/v solution of each) on fluorescence intensity of PTB (40 ngmL^−1^).

Various organized media were tested to investigate their effect on FI of PTB with the addition of 0.5 mL of their individual aqueous solutions to PTB solution. Various surface active agents including tween 20, 80, and 85, Cremophor El, Cr RH 40 (nonionic surface active agents) sodium dodecyl sulfate (SDS) (anionic surface active agent), carboxymethylcellulose (CMC), and macromolecules such as *β*-cyclodextrin were tried. The greatest FI was acquired utilizing Cr RH 40 and tween 20 as displayed in Figure 3. Commonly, nonionic surface active agents possess superior solubilization power for hydrophobic drugs than ionic surface active agents. This may be due to their, relatively, lower critical micelle concentration (cmc) values [22]. Nevertheless Cr RH 40 was used in our study due to its low fluorescence intensity at the analytical wavelength when compared with other surface active agents such as tween 20.

#### 3.2.2. Influence of Cr RH 40 Volume

**Figure 4:**
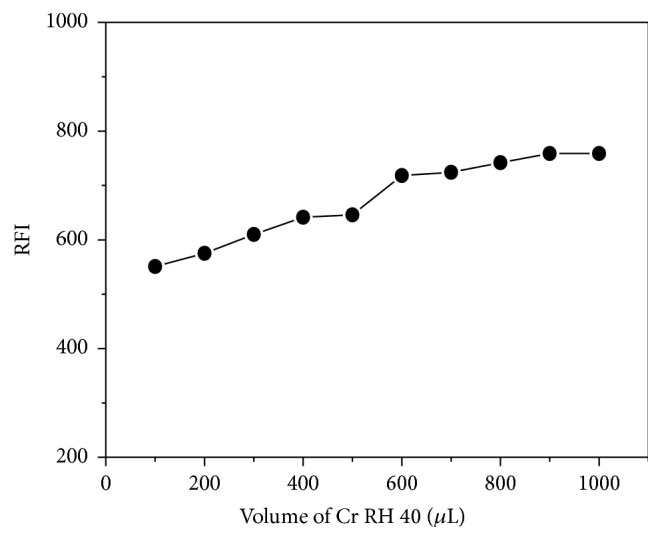
Effect of Cr RH 40 volume (1% w/v) on fluorescence intensity of PTB (40 ngmL^−1^).

The influence of Cr RH 40 on the fluorescence intensity was demonstrated using various volumes of 1% w/v Cr RH 40. It was clear from Figure 4 that increasing volumes of Cr RH 40 solution led to corresponding increase in PTB fluorescence intensity up to 0.6 mL (1% w/v). After this volume, no more increment in fluorescence intensity was detected. Therefore 0.7 mL 1% w/v Cr RH 40 solution was selected as the optimum volume for PTB determination (Figure 4).

#### 3.2.3. Influence of pH

**Figure 5:**
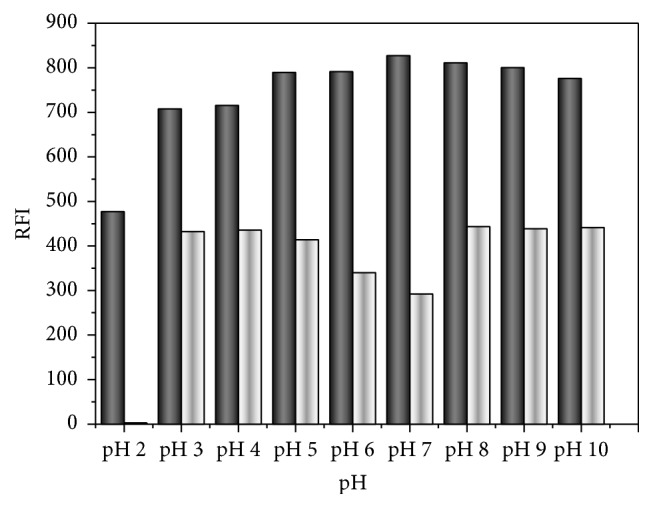
pH effect on FI of 40 ngmL^−1^ PTB in 0.5 mL 1%, w/v Cr RH 40 in acetonitrile (black column in presence of Cr RH 40 and white column in absence of Cr RH 40).

pH influence on FI of PTB was tested utilizing various buffers that cover pH range of 2–10, for instance, pH range 2–7 was covered using 0.1 M phosphate buffer while pH range 8–10 was covered using 0.1 M borate buffer. The data showed an initial increase of FI as the pH increased and highest FI was reached at pH 7.0 ± 0.2 (Figure 5). This behavior may infer the instability (hydrolysis) of Cr RH 40 triricinoleate ester at acidic and basic media.

#### 3.2.4. Influence of Diluting Solvent

**Figure 6:**
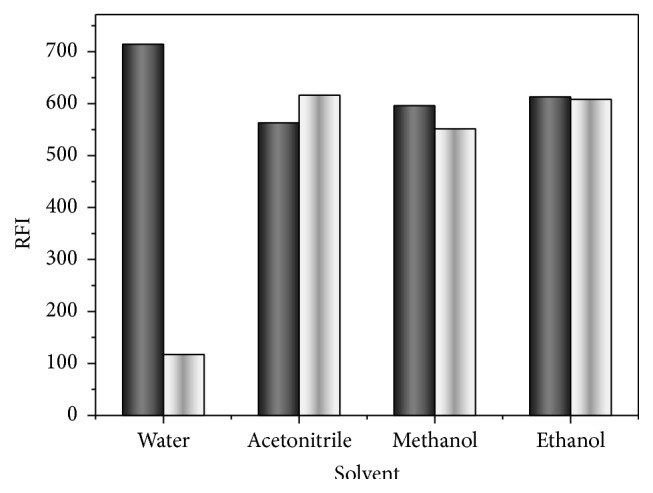
Effect of the diluting solvent on fluorescence intensity of PTB (40 ngmL^−1^); black column in presence of Cr RH 40; and white column in absence of Cr RH 40.

To investigate the effect of different diluting solvents, water, methanol, ethanol, and acetonitrile were used. Water showed the maximum FI compared to the others, which can be due to the variation in polarity of the medium that may have led to physical interaction between the studied solvents and PTB excited singlet state (Figure 6). Accordingly, dilution all over the study was performed using water. Reduction of FI of PTB in Cr RH 40 in presence of methanol, acetonitrile or ethanol, can be attributed to the denaturation of the micelles. Methanol or ethanol, as short chain alcohols, is mostly dissolved in the aqueous phase and alters the properties of the solvents which influences the formation of the micelle. Moreover methanol or ethanol may reduce micellar size and may decompose the surface active agent aggregate at high concentration [23].

#### 3.2.5. Influence of Time

Time influence on the stability of PTB FI in Cr RH 40 was tested. PTB FI produced immediately and persisted for one hour at least.

From the above experimental procedures, it was clear that maximum response was obtained by addition of 0.7 mL of Cr RH 40 and one mL of phosphate buffer to PTB aliquots and using water as a diluting solvent then recording RFI at 457 nm (after excitation at 305 nm).

## 4. Method Validation

Different validation parameters such as linearity, sensitivity, accuracy, specificity, repeatability, and reproducibility were calculated according to EMA guidelines for validation of the bioanalytical method [24].

### 4.1. Linearity and Calibration Range

Calibration plot of PTB quantitation was created by drawing FI versus PTB nominal concentration. The graph was linear over the concentration range listed in Table 1. Data resulting from statistical analysis [25] were also anticipated in Table 1 showing high determination coefficient values (*r* ^2^) and low standard deviation values of the residuals (*S*_*y*/*x*_), slope (*S*_*b*_) and intercept (*S*_*a*_) as well as low % RSD and % error. The linearity of PTB calibration plot was also verified by these values.

### 4.2. Limit of Quantitation (LOQ) and Limit of Detection (LOD)

Limit of quantitation (LOQ) and limit of detection (LOD) were calculated using ICH Q2 (R1) guidelines [24]. Calculation of LOQ is based on finding the lowest measurable concentration of PTB below which the calibration plot is deviated from linearity while the LOD calculation is based on estimating the minimal readily detectable PTB concentration. The data are abridged in Table 1. LOQ and LOD values were computed in accordance to the following formulae:

(1) LOD=3.3σm,LOQ=10σm,

where *σ* and *m* represent SD of the intercept and the slope of regression line, respectively. The literature published data [26] (maximum plasma concentration (*C*_max⁡_) of PTB around 50 ngmL^−1^) revealed that our LOQ (2.742 ngmL^−1^) is considerably lower than PTB *C*_max⁡_ and, consequently, PTB can be easily determined in plasma.

### 4.3. Accuracy and Precisions

Tables 2 and 3 display intra- and interday precisions and accuracy of the suggested method. Three replicate samples for 4 various concentrations of PTB were analyzed in the same day and in three consecutive days to calculate intra- and interday precision, respectively. For calculation of accuracy (as % bias) the following equation was followed:

(2) $$\begin{array}{c} \%\;\text{Bias}=\frac{\left(\text{Supposed PTB concentration}-\text{measured PTB average concentration}\right)}{\text{Supposed PTB concentration}}*100. \end{array}$$

The range of % bias was −3.6 to 2.16%, stating the method accuracy. The intra- and interday precisions were described as % recovery. Mean recovery (around 100%) and low values of RSD are evidences for the inter- and intraday precision of the adopted procedures (Table 3). All these results indicate that the accuracy and the precision of the adopted spectrofluorimetric method.

### 4.4. Robustness

Robustness of the method was examined by assessing the susceptibility of determinations to small modifications of the analytical conditions. It was clear from Table 4 that deliberate changes which may occur throughout the experimental conditions that have not altered PTB FI.

### 4.5. Selectivity

Selectivity of the method was demonstrated by the analysis of PTB in different biological matrices such as plasma and urine. It was clear from Table 6 that the proposed method is selective enough for PTB determination in these matrices (as indicated by the small values of SD for PTB analysis in plasma and urine), and there were no interferences from urine or plasma endogenous components.

### 4.6. Stability and Dilution Integrity

PTB stability in urine and plasma samples was evaluated through the analysis of three replicates of samples at two different concentrations subjected to various processing and storage conditions. Three portions of the individual samples were used for evaluation of the bench-top stability (short term stability), freeze thaw stability, and long term stability. Bench-top stability was assessed after exposure of the spiked plasma and urine samples to room temperature for at least 6 h. The freeze thaw stability was evaluated after three freeze (at around −80°C) thaw (room temperature) cycles. Long-term stability was assessed after storage of the spiked plasma and urine samples at around −80°C for 14 days. The stability of PTB working and stock solutions was evaluated at room temperature for 24 h and at 2–8°C for 14 days. All stability studies were carried out against freshly spiked calibration standards. The samples were considered stable in plasma and urine if the deviation from the mean calculated concentration of stability quality control samples was within ±15%. Dilution integrity exercise was also performed to ensure the integrity of PTB in samples which are above upper limit of the calibration range and need to be diluted. A fresh stock PTB solution was prepared and spiked in plasma and urine to give a conc. level of 1.8 times of the highest concentration in the calibration range; it was then diluted 2 and 4 times. Three aliquots of both dilutions were analyzed and the integrity of the samples was considered to be maintained if % nominal is within ±15% of nominal values and % CVs ≤ 15% at both diluted levels. All the stability studies results were summarized in Table 5. It was clear that stock and working solutions of PTB were stable at room temperature for 24 h and at 2–8°C for at least 14 days. PTB was stable in human plasma and urine at room temperature and at −80°C. The mean recovery % and CV % for 1/2 and 1/4 dilution samples were within 95–105% and <1.4%, respectively.

## 5. Applications

### 5.1. Analysis of PTB in Human Plasma

The high sensitivity of the adopted analytical procedure demonstrated that PTB can be easily quantified in human plasma. PTB is an orally active drug and its *C*_max⁡_ is reached after administration by 5-6 hours [27]. The *C*_max⁡_ of PTB ranged from 50 to 77 ngmL^−1^ [26, 28]. Consequently, the level of PTB in plasma fell into in the linearity range of the adopted procedure (Table 1). Table 6 revealed that the mean absolute recoveries and the % RSD of PTB in plasma samples are 0.74% and 85.01%, respectively. The poor recovery of PTB (below 90%) may be attributed to its high plasma protein binding (>99% in humans).

### 5.2. Analysis of PTB in Urine

Almost 1% of PTB daily recommended dose (around 45 mg) is excreted in urine as it is [28]. Consequently, the drug level in urine (0.45 *µ*gmL^−1^) is higher than the working range of the adopted method by 100 times. The results stated in Table 5 revealed that mean absolute recoveries and % RSD of PTB in spiked urine samples are 2.16% and 98.77%, respectively. These excellent results (around 100% mean absolute recovery) may be revealed by the action of the big dilution (100 fold) that was necessary to reach our working range (5–120 ngmL^−1^). This vast dilution led to minimizing the interferences that emerged from the endogenous amino acids.

### 5.3. Postulated Mechanism of Cr RH 40 Enhancement

The improved PTB fluorescence may be due to either a rise in the quantum yield and/or an enhanced absorption at the excitation wavelength (*λ*_ex_). Molar absorptivity calculation of PTB in Cr RH 40 took place at 305 nm (*λ*_ex_). The *ε*_micellar_/*ε*_acetonitrile_ ratio was almost equal to one which means that the PTB fluorescence enhancement is not due to increase of PTB in micellar system at its *λ*_ex_. PTB quantum yield was 0.879 in acetonitrile and 0.946 in the existence of Cr RH 40. This increase in PTB quantum yield in micellar solution can be a result of the protection of the lowest excited singlet state from nonradiative processes in Cr RH 40. Calculation of the quantum yield was performed by applying the following equation [11]:

(3) $$\begin{array}{c} \phi d=\phi q\frac{Fd}{Fq}\cdot \frac{Aq}{Ad}, \end{array}$$

where *ϕd* is the PTB fluorescence quantum yield, while *ϕq* is the quinine fluorescence quantum yield. *Fd* and *Fq* are the integral FIs of the PTB and quinine, respectively; *Ad* and *Aq* are the absorbance values of PTB and quinine at excitation wavelength, respectively. To diminish the inner effect error, the PTB concentration was selected as to produce absorbance below 0.05 [29].

## 6. Conclusions

This study represents the first analytical procedure for PTB quantification with LOD and LOQ values of 0.905 and 2.742 ngmL^−1^, respectively. These values ensured the higher sensitivity of the adopted method. This method can be considered among the green analytical methodologies, because of the absence of organic solvents in the procedure. The adopted method is efficient, rapid, and simple compared to conventional chromatographic techniques such as HPLC. The proposed method could be applied for the routine analysis of PTB in human plasma as well as human urine by virtue of its practical simplicity and sensitivity.

## Figures and Tables

**Table 1:** Analytical performance data for the spectrofluorimetric quantitation of PTB.

Parameter	PTB
Wavelength [*λ*_ex_/*λ*_em_] (nm)	305/457
Linearity range (ngmL^−1^)	5–120
Intercept (*a*)	20.59
Slope (*b*)	17.72
Determination coefficient (*r* ^2^)	0.9992
SD of residuals (*S*_*y*/*x*_)	19.98
SD of intercept (*S*_*a*_)	4.859
SD of slope (*S*_*b*_)	0.07191
% RSD^a^	1.048
% error^b^	0.433
LOD (ngmL^−1^)^c^	0.905
LOQ (ngmL^−1^)^d^	2.742

^a^Percentage relative standard deviation for six replicate samples.

^b^Percentage relative error for six replicate samples.

^c^Limit of detection.

^d^Limit of quantitation.

**Table 2:** Accuracy of the spectrofluorimetric method for determination of PTB.

Days	Actual conc. (ngmL^−1^)	Mean conc. (ngmL^−1^)	±SD	% RSD	% bias	SEM
1	20	19.60	0.34	1.74	−1.99	0.15
2	20	19.29	0.22	1.16	−3.6	0.09
3	20	19.57	0.74	3.78	−2.17	0.33

1	60	60.89	0.36	0.6	1.47	0.16
2	60	59.72	0.53	0.89	−0.47	0.22
3	60	59.59	0.72	1.21	−0.69	0.32

1	80	81.75	0.77	0.95	2.16	0.35
2	80	80.92	0.86	1.06	1.15	0.35
3	80	79.32	1.31	1.65	−0.85	0.59

1	120	120.26	1.19	0.99	0.21	0.53
2	120	118.38	1.04	0.88	−1.36	0.43
3	120	119.73	0.65	0.54	−0.22	0.29

SEM: standard error of the mean; 1, 2, and 3 represent measurements obtained on day 1, day 2, and day 3, respectively (*n* = 3 for each day).

**Table 3:** Intra- and interday precision and accuracy for PTB determination by the adopted analytical method.

Nominal conc. (ngmL^−1^)	Intraday		Interday	
	Calculated conc. (ngmL^−1^)	Recovery (% ± RSD)^a^	Calculated conc. (ngmL^−1^)	Recovery (% ± RSD)^a^
20	19.6	98.00 ± 2.01	19.47	97.35 ± 0.82
60	60.89	101.48 ± 0.69	59.97	99.95 ± 1.37
80	81.75	102.19 ± 1.09	80.5	100.63 ± 1.88
120	120.26	100.22 ± 1.14	119.46	99.55 ± 1.81

^a^Mean of three determinations.

**Table 4:** Robustness results of the proposed analytical procedure.

Variation of the experimental parameters	% Recovery ± SD^a^
No change^b^	99.68 ± 0.88
Cr RH 40 volume (*µ*L)	
580	101.98 ± 0.39
620	101.83 ± 1.16
pH	
7.8	101.76 ± 0.84
8.2	103.00 ± 0.35
Buffer volume (*µ*L)	
0.95	102.37 ± 0.98
1.05	102.59 ± 1.12
Temperature (°C)	
20	102.61 ± 0.35
30	99.68 ± 1.17

^a^Average of triplicate measurements.

^b^According to the general calibration procedures.

**Table 5:** Stability and dilution integrity data of PTB.

Stability	Nominal conc. (ngmL^−1^)	Plasma			Urine		
		Measured conc. (ngmL^−1^ ± SD)	Precision (CV %)	Accuracy (*R* %)	Measured conc. (ngmL^−1^ ± SD)	Precision (CV %)	Accuracy (*R* %)
Bench top (6 h)	50	46.01 ± 0.42	0.913	92.01 ± 0.84	47.67 ± 0.40	0.848	95.35 ± 0.81
	100	92.08 ± 0.68	0.736	92.08 ± 0.68	95.63 ± 1.53	1.605	95.63 ± 1.53

Freeze thaw (3 cycles)	50	50.67 ± 0.31	0.606	101.34 ± 0.61	50.00 ± 1.00	2.016	100.0 ± 2.02
	100	100.47 ± 1.08	1.071	100.47 ± 1.08	100.0 ± 1.07	1.074	100.0 ± 1.0

14 days at −80°C	50	42.94 ± 0.91	2.108	85.87 ± 0.91	47.20 ± 0.953	2.019	94.40 ± 1.91
	100	86.67 ± 0.68	0.788	86.67 ± 0.68	94.11 ± 1.26	1.343	94.11 ± 1.26

Dilution integrity	45	43.54 ± 0.61	1.393	96.76 ± 1.35	44.48 ± 0.59	1.337	98.84 ± 1.32
	90	90.21 ± 1.66	1.835	100.23 ± 1.84	88.27 ± 0.73	0.824	98.08 ± 0.81

**Table 6:** Results of PTB determination in its pure form, human plasma, and human urine samples.

	Pure form			Plasma samples			Urine samples		
	Amount taken (ngmL^−1^)	Amount found (ngmL^−1^)	% found	Amount added (ngmL^−1^)	Amount found (ngmL^−1^)	% found	Amount added (ngmL^−1^)	Amount found (ngmL^−1^)	% found
Parameter	20	19.6	98.02	50	42.5	85.00	10	9.94	99.42
	60	60.89	101.48	60	50.49	84.15	20	19.99	99.95
	80	81.75	102.19	70	59.44	84.91	60	60.09	100.15
	120	120.26	100.21	75	64.47	85.96	120	114.67	95.56

Mean			100.48			85.01			98.77

±SD			1.83			0.74			2.16
